# Visual perception of landscape renovation in Qinyang’s historical and cultural streets: evidence from eye tracking of static streetscape images

**DOI:** 10.3389/fpsyg.2026.1848298

**Published:** 2026-09-04

**Authors:** Weiwei Zhang, Bocheng Wang, Lei Feng, Xuechun Wang, Yichuan Zhang, Lifang Qiao

**Affiliations:** 1 School of Urban Design, Henan Technical College of Construction, Zhengzhou, China; 2 School of Horticulture and Landscape Architecture, Henan Institute of Science and Technology, Xinxiang, China

**Keywords:** AOI, eye tracking, historical and cultural streets, landscape renovation, semantic segmentation, static streetscape images, visual perception

## Abstract

Using eye-tracking technology, this study examined how landscape renovation schemes for historical and cultural streets (HCSs) influence public visual attention allocation under static streetscape image-viewing conditions. Ten HCS nodes in the central urban area of Qinyang were selected as the study sites. Paired pre-renovation field photographs and post-renovation renderings were used as stimulus materials, and participants’ eye-movement data during image viewing were collected using the HTC Vive Pro Eye system in conjunction with the ErgoLAB platform. The analysis first characterized the overall distribution of visual attention across streetscapes by means of fixation heat maps. At the area of interest (AOI) level, each streetscape was subdivided into 14 element categories, from which relevant eye-tracking metrics were extracted. In parallel, semantic segmentation was employed to derive visual structural indicators for cross-validation. Differences between pre- and post-renovation conditions were then tested using paired-sample t-tests (*p* < 0.05). The results showed that, following renovation, fixation heat maps exhibited a clear shift in visual hotspots from the forward roadway area toward the street-side interfaces and landscape elements. AOI analysis further indicated that, in most streetscapes, attention allocated to culturally relevant elements, such as historical buildings and historical signage, increased, while environmentally supportive elements, including vegetation, pedestrian interfaces, and public facilities, were simultaneously enhanced. By contrast, attention directed to visually disruptive elements, such as vehicles, obstructions, and cluttered commercial signage, generally declined. Nevertheless, a small number of streetscapes still exhibited competition for attention among elements and uneven improvement effects. Overall, the results indicate that, under static streetscape image-viewing conditions, observers allocated greater visual attention to elements associated with cultural expression, vegetation landscapes, and the pedestrian environment in the post-renovation images, showing an overall shift in visual attention patterns. However, because the pre- and post-renovation stimuli consisted of field photographs and design renderings, respectively, the results may have been influenced by differences in image types. Therefore, these findings should be interpreted as changes in visual perception under design-scheme representation conditions rather than direct evidence of effects in the actual built environment, and they should not be directly equated with real street walking experiences.

## 1 Introduction

Historical and cultural streets (HCSs) function not only as material repositories of urban memory but also as key settings for everyday mobility and public life. In recent years, rapid urban renewal and commercial transformation have improved environmental quality in many cities; yet these processes have also frequently weakened historical character, generated visually disordered street interfaces, and allowed traffic and commercial activity to obstruct the streetscape. As a consequence, the cultural information embedded in traditional streets has often become difficult to perceive clearly. A growing body of research in China and internationally has approached this issue from the perspective of human experience and has used quantifiable perceptual indicators to explain how street-environment elements shape attractiveness, willingness to linger, and place evaluation (Shao et al., 2023; Ma et al., 2025; Li et al., 2021). Historic and cultural street spaces are often required to strike a balance among cultural representation, everyday order, and commercial vitality. Whether their renovation can enhance the visual recognition of cultural information and the perception of environmental quality requires more direct quantitative evidence (Zube et al., 1982; Wartmann et al., 2021; Ma et al., 2021).

Recent studies on HCSs and heritage landscapes have further shown that eye tracking is highly compatible with psychological approaches to environmental perception because it can connect visible spatial elements with attentional allocation, cognitive processing, emotional response, and place-related evaluation. In research on historical and cultural districts, eye-tracking metrics and heat maps have been used to identify how different environmental elements are visually recognized and how subjective impressions correspond to objective gaze behavior (Jiang et al., 2025; Zheng et al., 2024). Similar evidence from traditional architectural heritage and historical landscapes indicates that culturally distinctive facades, architectural details, and symbolic elements tend to attract longer fixations and more frequent visual engagement, suggesting that eye movements can reveal the cognitive salience of heritage information (Deng et al., 2024; Hao et al., 2024). From the perspective of environmental psychology, the integration of eye tracking with semantic differential evaluation, scenic beauty estimation, or emotional assessment provides a means of linking visual attention with affective appraisal and preference formation. For example, studies of fortress heritage landscapes and architectural heritage tourism have demonstrated that eye-movement indicators can complement subjective psychological evaluations and help explain how heritage elements generate visual experience, positive emotions, and behavioral intentions (Xu et al., 2024; Li et al., 2025). These findings suggest that eye tracking is not merely a technical tool for recording gaze positions, but also an effective psychophysiological method for interpreting how observers perceive, evaluate, and emotionally respond to historic streetscapes.

Visual perception plays a central role in the formation of street experience (Gobster et al., 2019). Conventional questionnaire surveys and expert evaluations can capture overall preference, but they cannot reveal the process or mechanism of attentional allocation (Liu and Neisch, 2025). Eye-tracking technology can directly record fixation location, fixation count, total fixation duration, and time to first fixation, thereby providing objective measures of the intensity of visual processing and the pathways through which attention is directed toward streetscape elements. Recent studies have shown that eye tracking can capture observers’ patterns of visual engagement with street boundaries, interface details, and signage systems under controlled viewing conditions, while also revealing how street form, degree of commercialization, and spatial order influence the distribution of visual attention (Ma et al., 2024). In highly commercialized HCSs, advertisements, vehicles, and disordered shop signs often attract attention at an early stage and occupy substantial fixation resources, thereby diminishing the visibility of historical buildings and cultural nodes. Therefore, if the aim is to evaluate whether a renovation design has restructured fixation patterns at the level of image viewing, subjective judgment alone remains insufficient. Eye-tracking evidence is needed to determine whether observers’ attention, before and after renovation, becomes more strongly directed toward elements such as historical buildings, cultural signage, and pedestrian-friendly features (Fang et al., 2024; Qin et al., 2023; Yang et al., 2024).

Streetscape renovation is also typically accompanied by changes in visual-structural indicators, including the green view ratio (GVR), sky openness (SO), pedestrian-space proportion (PSP), spatial enclosure (SE), and the motorized traffic impact ratio (MTIR) (Biljecki and Ito, 2021). With the development of semantic segmentation and deep-learning techniques for streetscape imagery, these indicators can now be extracted at the pixel level and used to explain streetscape quality, pedestrian vitality, and landscape preference (Ito et al., 2024; Li et al., 2023). For example, studies based on Google Street View imagery and computer vision have shown that street greenery and the GVR are significantly associated with walking time, physical activity, and perceived environmental quality, and semantic segmentation has also been used to construct quantitative models of street vitality and walkability (Li et al., 2015; Zhang F. et al., 2024). More recent work has further combined deep-learning-based element recognition with eye-tracking data to explain how specific environmental elements become visual foci and, in turn, shape ecological esthetics and overall preference (Zhang T. et al., 2024; Xia et al., 2021a; Xia et al., 2021b; Ye et al., 2019). Therefore, by integrating the changes in element composition derived from semantic segmentation with the attention shifts revealed by eye-tracking data, it becomes possible to evaluate whether the renovation scheme has enhanced the visual recognizability of cultural information at the level of static streetscape image viewing (Wang et al., 2019; Kang et al., 2023; Wang et al., 2022; Ma et al., 2023; Gao et al., 2025).

This study focuses on the renewal of HCSs in the central urban area of Qinyang. Ten representative streetscape images were selected to establish the pre-renovation baseline scenarios. Renovation schemes were then developed, and corresponding post-renovation renderings were generated for each original scene. Finally, pre-renovation field photographs and post-renovation renderings were used as paired stimuli. Eye-tracking metrics were integrated with visual-structural indicators derived from semantic segmentation for cross-validation, thereby providing an interpretable and comparable image-perception evaluation framework for historical and cultural street renovation. It should be noted that this study employed a static streetscape image-viewing experiment, and the findings primarily reflect differences in visual attention allocation between pre- and post-renovation streetscape images under controlled experimental conditions. Since the pre- and post-renovation stimuli were, respectively, field photographs and design renderings, the two image types may differ in lighting, color saturation, image cleanliness, visual noise, and the representation of dynamic elements such as vehicles and pedestrians. Therefore, the results of this study cannot be fully equated with the comprehensive experience of walking through actual streets, nor can they be directly regarded as causal evidence of renovation effects in the real built environment (Hartig et al., 2003; Jahani and Saffariha, 2020; Xu et al., 2023).

## 2 Study area and methods

### 2.1 Overview of the study area

**Figure 1:**
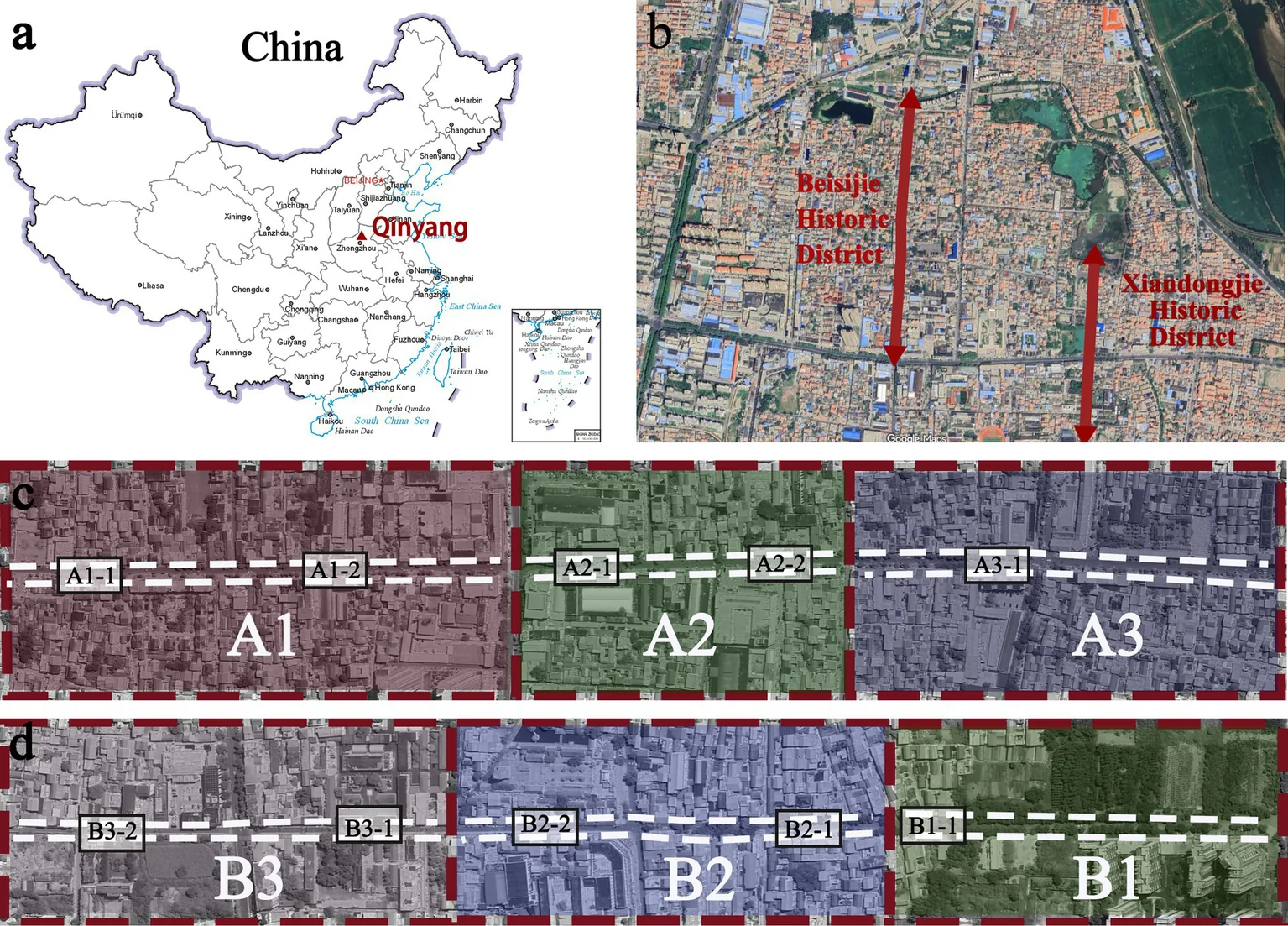
Location of the study area [**(a)** Location map of Qinyang City; **(b)** Location map of the HCSs; **(c)** Location map of the renovated streetscape sample sites in the Beisi HCS; **(d)** Location map of the renovated streetscape sample sites in the Xiandong HCS].

The study area was located in the central urban district of Qinyang, in northwestern Henan Province. Qinyang is a historic city with a recorded history extending back more than one millennium and with a rich cultural legacy. As interfaces between everyday urban life and the public representation of place, the HCSs in the central city serve circulation, commerce, and public activity while also preserving historical character, displaying cultural symbols, and transmitting place memory. At present, however, the legibility of historical and cultural information remains in tension with pervasive visual disorder. In some segments, aging street interfaces, disordered overhead wires and ancillary facilities, the encroachment of vehicles and mobile vendors into pedestrian space, and the mismatch between commercial signage and façade style have obscured historical buildings and cultural signage or rendered them insufficiently legible. Two major streets (Beisi HCS and Xiandong HCS) were selected as the study areas (Figure 1).

### 2.2 Sample selection

**Figure 2:**
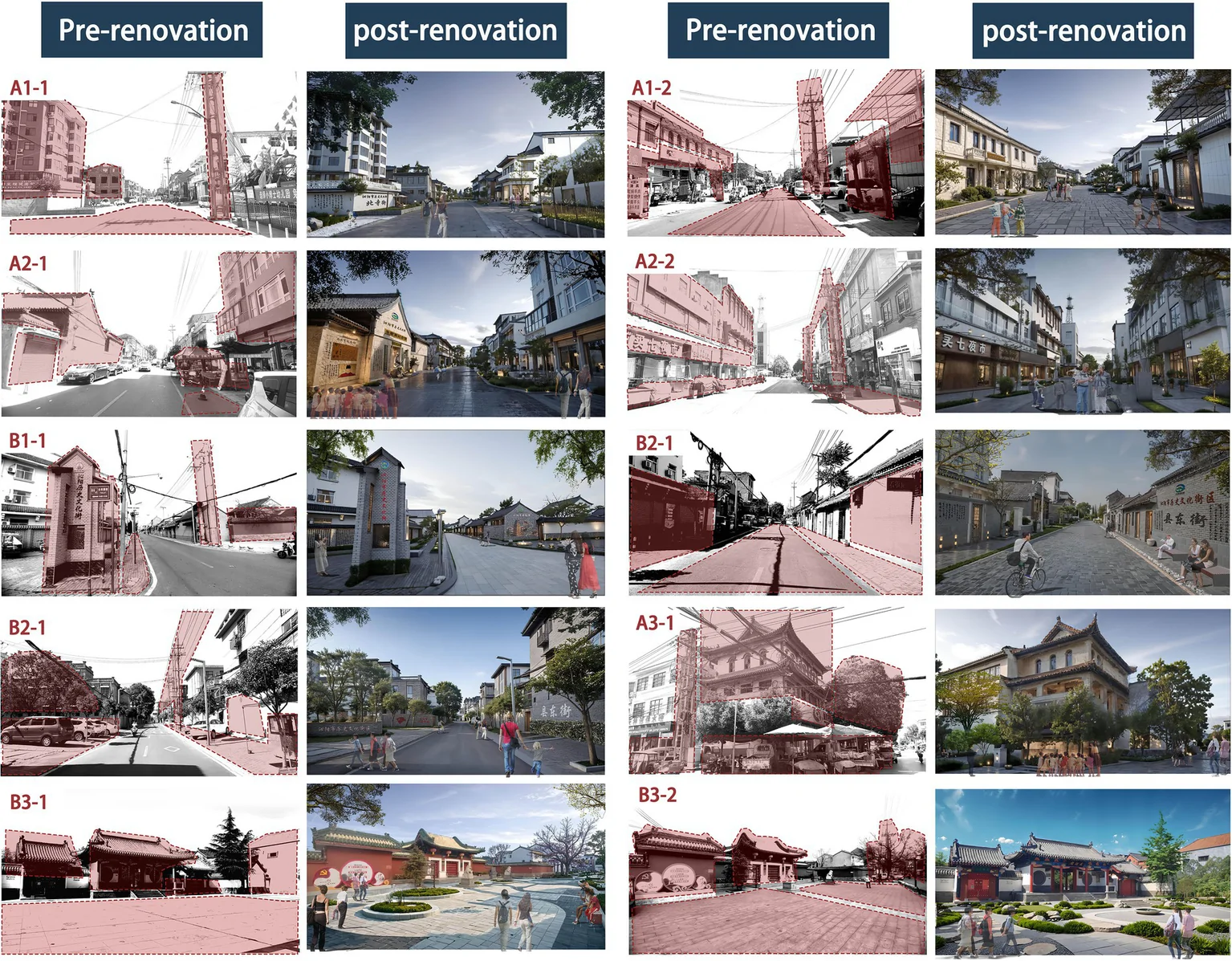
Pre- and post-renovation images of typical streetscape nodes.

On the basis of preliminary eye-tracking observations and the judgments of five experts in landscape architecture and urban–rural planning, ten typical streetscape nodes in the central urban district were selected as sample sites because their spatial quality was considered weak and in need of renovation (Figure 2). These nodes were used to construct paired pre- and post-renovation stimuli. The renovation schemes were designed to highlight historical building interfaces, introduce cultural wayfinding and signage nodes, optimize planting composition, and control or attenuate negative elements such as obstructions, vehicles, and street vendors. Each pre-renovation photograph and corresponding post-renovation rendering of each node constituted a paired sample, with the viewing angle, composition, and major spatial structures kept as consistent as possible. It should be noted that the pre-renovation images were field photographs, whereas the post-renovation images were design renderings. The two types of images may differ in lighting, color saturation, image cleanliness, visual noise, and the representation of dynamic elements such as vehicles and pedestrians. Therefore, the comparisons between pre- and post-renovation images in this study were primarily intended to analyze the changing trends in visual attention patterns under the conditions of design-scheme representation, and the observed differences in eye-tracking behavior should not be entirely attributed to the renovation interventions themselves.

### 2.3 Participant recruitment

A total of 51 participants were recruited for the experiment. All participants had normal or corrected-to-normal vision and reported no color weakness, color blindness, or other physiological impairments that might affect eye-tracking performance. The participants were drawn primarily from Henan Institute of Science and Technology and related groups, including 26 males (50.98%) and 25 females (49.02%). In terms of age distribution, 26 participants were aged 20–25 years (50.98%), 13 were aged 25–30 years (25.49%), 8 were aged 31–40 years (15.69%), and 4 were aged 41 years or older (7.84%). With respect to educational background, 21 participants held a bachelor’s degree (41.18%), 20 held a master’s degree (39.22%), 5 held a doctoral degree (9.80%), and 5 were non-student participants (9.80%). Overall, the sample was composed predominantly of young, highly educated individuals, while also including a small number of middle-aged and non-student participants. Prior to the experiment, all participants were informed of the study purpose, experimental procedure, intended use of the data, and their right to withdraw at any time. Participation was entirely voluntary and based on fully informed consent, and all participants signed a written informed consent form before the experiment. The collected data were used exclusively for academic research and were anonymized during subsequent processing and analysis.

It should be noted that the sample in the present study remained dominated by young, highly educated individuals. Although a small number of participants over 31 years of age and several non-student participants were included, the sample still exhibited a degree of concentration in terms of age, geographic background, and occupational profile. Therefore, the results of this study primarily reflect the visual perception characteristics of this participant group under static streetscape image-viewing conditions, and cannot yet be directly generalized to represent all user groups of historical districts, including older residents, local residents, street-front businesses, non-local visitors, and families with children.

### 2.4 Eye-tracking experiment

#### 2.4.1 Eye-tracking procedure

The experiment was conducted using the HTC Vive Pro Eye system, and eye-movement data were acquired and synchronously recorded through the ErgoLAB human–environment synchronization platform. It should be noted that the experimental stimuli were presented as two-dimensional static streetscape images, without bodily movement, route selection, or interactive VR navigation. To enhance data stability and comparability, the experiment was carried out in a closed and quiet indoor environment. Key conditions, including lighting, headset fit, and stimulus presentation parameters, were standardized throughout the experiment in order to minimize the influence of external interference on participants’ visual behavior and to ensure that all participants were tested under identical viewing conditions (Xu et al., 2024).

A total of 20 stimulus images were used in the pre- and post-renovation comparison experiment, comprising 10 pre-renovation field photographs and 10 corresponding post-renovation renderings. Each participant viewed all 20 stimulus images. The experiment adopted an individual testing protocol, such that only one participant entered the experimental setting and completed the viewing task at a time; thus, no image was viewed simultaneously by multiple participants. To ensure consistency in stimulus exposure, each streetscape image was presented for 15 s, with a 3-s blank screen inserted between successive images as a visual buffer to reduce carryover effects from the preceding image. Before the formal experiment began, the researchers first explained the experimental procedure and precautions to the participants and allowed a brief period for adaptation to the device, during which any discomfort symptoms, such as dizziness or nausea, were monitored. Only after confirming that the participant could comfortably adapt to the device was the formal testing session initiated. Eye-tracking calibration was completed prior to the formal experiment to ensure accurate recording of ocular movement data. Once the experiment commenced, the streetscape stimuli were presented in random order, and care was taken to avoid the consecutive presentation of the pre-renovation and post-renovation images corresponding to the same site. The ErgoLAB platform synchronously recorded the participants’ eye-movement data throughout the viewing process.

#### 2.4.2 AOI definition

To identify differences in the allocation of visual attention to distinct streetscape components, areas of interest (AOI) were defined for each streetscape image in the present study. AOI annotation was performed in the ErgoLAB human–environment synchronization platform, and element boundaries were determined through a combined procedure of manual delineation and independent review. The AOIs were classified on the basis of the spatial composition of HCSs, the findings of the preliminary field investigation, and the streetscape element classification framework established in the broader dissertation. With reference to four dimensions—environmental landscape, historical and cultural features, commercial/living functions, and spatial structure—the streetscape images were divided into 14 AOI categories: sky, vegetation, vehicular roadway, residential buildings, historical buildings, sidewalks, commercial signage, obstructions, facilities, commercial buildings, vehicles, historical signage, paving, and commercial activities. According to the preliminary survey data, six categories—vehicles, commercial buildings, obstructions, commercial signage, residential buildings, and vehicular roadways—were classified as negative elements in the context of historic and cultural district renovation. It should be noted that, across the ten selected sites, the residential buildings were characterized by stylistic disorder and were markedly inconsistent with the historical and cultural landscape context, while the vehicular roadways appeared visually chaotic; accordingly, both residential buildings and vehicular roadways were treated as negative elements. The remaining eight categories were classified as positive elements. AOI boundaries and category assignments were independently reviewed by two researchers, and any discrepancies in boundary delineation were resolved through discussion. Cohen’s kappa coefficient and percentage agreement were used to quantify annotation consistency (Appendix A).

#### 2.4.3 Semantic segmentation and visual-structural indicators

To further quantify the impact of street renovation on the spatial environment, semantic segmentation analysis was performed on both the pre-renovation field images and the post-renovation renderings. The semantic segmentation procedure was conducted using the AutoDLAI platform. Specifically, the Cityscapes dataset configuration and the Mask2Former semantic segmentation model were selected within the platform to perform pixel-level classification of the streetscape images. The Cityscapes dataset is primarily designed for semantic understanding of urban street scenes and is well suited to identifying fundamental streetscape elements such as roads, buildings, sky, vegetation, vehicles, and pedestrian spaces. Mask2Former, in turn, provides strong general-purpose image segmentation capability and is suitable for multi-class pixel-level segmentation in complex streetscape images. Accordingly, this combination was considered sufficient to meet the basic requirements for extracting visual structural indicators from streetscape images in the present study.

The output of semantic segmentation enabled the pixels in each streetscape image to be classified into categories such as sky, buildings, roads, vegetation, vehicles, pedestrian spaces, and other environmental elements. On this basis, the pixel area of each element category was calculated, and a series of visual structural indicators were derived, including GVR, SO, PSP, SE, and MTIR. It should be noted that the Cityscapes label system is primarily intended for the recognition of basic urban street elements and cannot directly distinguish elements characterized by local cultural semantics or fine-scale visual features, such as historical buildings, historical signage, commercial signage, and obstructions. Therefore, the platform-generated segmentation results were further subjected to manual verification, with particular attention paid to regions prone to confusion, including historic versus ordinary buildings, roads versus sidewalks, vegetation boundaries, vehicles, and occluding objects. Regions showing obvious misclassification or substantial boundary deviation were corrected through manual interpretation, and the revised pixel classification results were used as the basis for subsequent indicator calculation. The calculation methods for the key visual structural indicators—namely, PSP, MTIR, SE, GVR and SO—are presented in Equations (1–5).

The target ranges of the visual and spatial indicators adopted in this study (GVR: 20–25%; SO: 30–50%; PSP: 30–40%; SE: 50–60%; MTIR: <10%) were determined based on a comprehensive consideration of domestic historical-district renovation practices and quantitative evaluation studies (Huang et al., 2024).

In the evaluation of pedestrian environments in HCSs, a study of the Tongwen area of Zhongshan Road in Xiamen demonstrated that the green view ratio significantly influences pedestrian visual perception and perceived comfort within historical districts (Li and Huang, 2020). Research on the visual quality evaluation of street spaces has established an assessment framework incorporating quantitative indicators such as SO and SE, which can be used to analyze the visual permeability and scale relationships of spatial forms in historical districts (Chen et al., 2025). Studies on pedestrian perception evaluation based on streetscape images have indicated that pedestrian-space proportion is closely associated with pedestrian friendliness, while street interface continuity and the built environment jointly determine walking experience, suggesting the need to increase the provision of pedestrian space. In historical-district renewal practices, studies of typical areas such as Enning Road in Guangzhou have shown through spatial perception approaches that environmental renovation can enhance spatial visual perception, highlighting the importance of controlling spatial scale and openness for improving experiential quality (He and Wang, 2019).


$$\text{Pedestrian}-\text{space proportion}=\frac{S\;\text{sidewalk}}{S\;\text{road}}$$ (1)

S sidewalk represents the area occupied by sidewalks within the street interface, whereas S road represents the area occupied by carriageways within the street interface, including both motor vehicle lanes and non-motor vehicle lanes.


$$\text{Motorized traffic impact ratio}=\frac{S\;\text{bicycle}+\text{rider}+\text{motorcycle}}{S\;\text{total}}$$ (2)

S bicycle + rider + motorcycle represents the sum of the pixel areas of non-motor vehicles in the street scene, whereas S total represents the total area of the street interface.


$$\text{Spatial enclosure}=\frac{S\;\text{building}+\text{wall}+\text{fence}+\text{pole}}{S\;\text{total}}$$ (3)

S building + wall + fence + pole represents the total area of physical enclosure elements within the street interface, whereas S total represents the total area of the street interface.


$$\text{Green view ratio}=\frac{S\;\text{vegetation}+\text{terrain}}{S\;\text{total}}$$ (4)

S vegetation + terrain represents the area of green-view elements within the street interface, whereas S total represents the total area of the street interface.


$$\mathrm{Sky}\;\text{openness}=\frac{S\;\mathrm{sky}}{S\;\text{total}-\text{vegetation}}$$ (5)

S sky represents the area of the sky within the street interface, whereas S total − vegetation represents the total area of the street interface after excluding vegetation.

### 2.5 Data analysis

Analysis of the visual-structural indicators of the streetscape images before and after renovation revealed changes in PSP, MTIR, SE, GVR and SO. These variations indicate that the visual composition and structural characteristics of the post-renovation images were altered to some extent. The professional background of the participants exerted only a limited influence on the mean values of these indicators; therefore, the data were analyzed in aggregate. To control for the influence of AOI area differences on fixation-based metrics, an area-standardization approach was adopted. Specifically, the pixel proportion occupied by each AOI within the image was first calculated as the AOI area ratio, after which fixation count and total fixation duration were each divided by this ratio to obtain fixation count density and fixation duration density, respectively. These area-standardized metrics make it possible to distinguish between increases in fixation attributable to post-renovation expansion of AOI area and those resulting from enhanced attractiveness per unit area, thereby providing a more accurate representation of the extent to which different elements attract visual attention. The differences in indicators before and after renovation were initially described using paired mean comparisons. The analytical framework can be further extended by incorporating paired-sample t-tests, false discovery rate correction, and Cohen’s dz. effect size estimation to quantify changes in visual attention allocation under pre- and post-renovation image-viewing conditions.

## 3 Results and analysis

### 3.1 Changes in fixation heat maps before and after streetscape renovation

**Figure 3:**
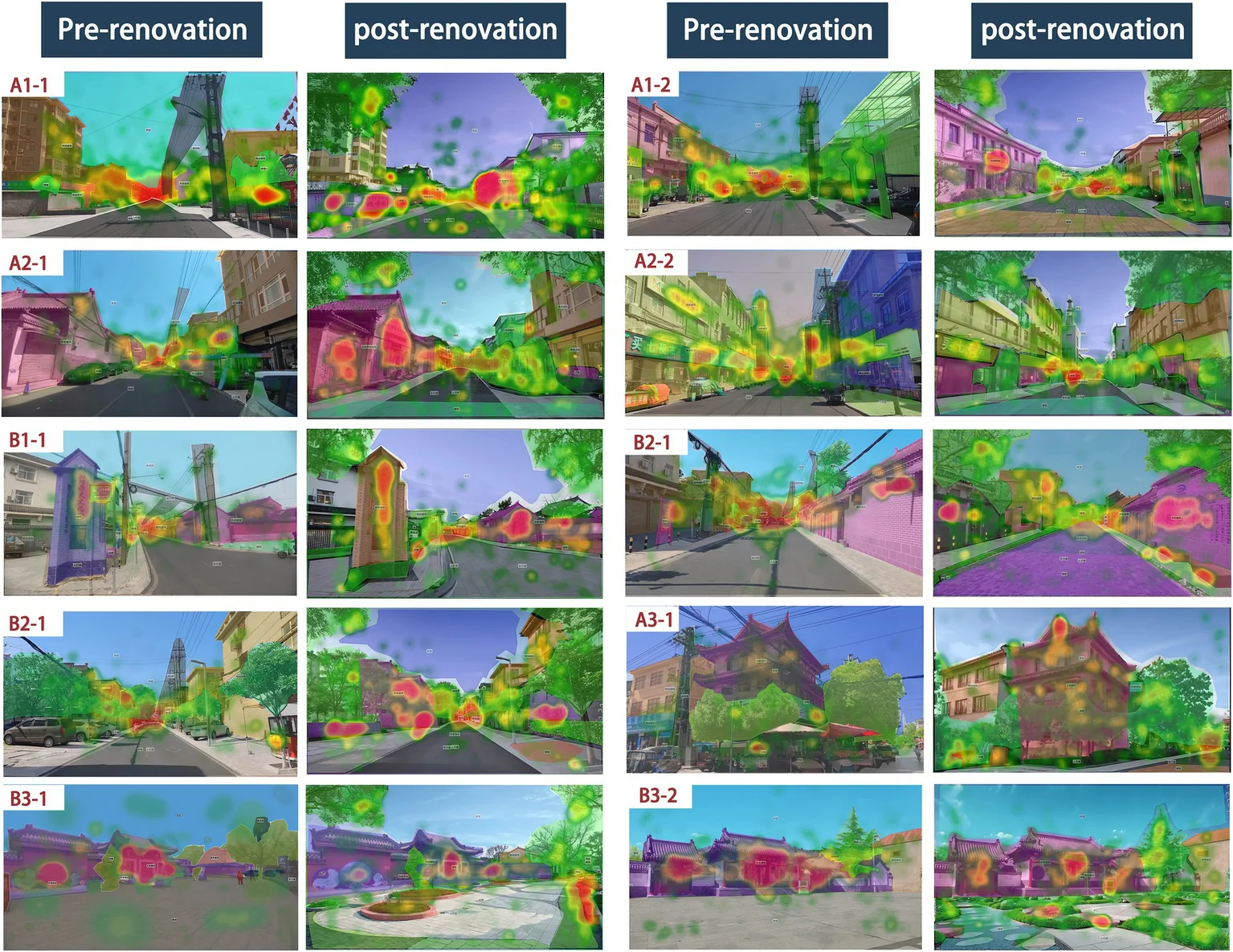
Fixation heat maps of typical streetscape nodes before and after renovation.

Comparison of fixation heat maps between pre- and post-renovation streetscapes showed that (Figure 3): in the post-renovation images, participants’ gaze distribution shifted from being predominantly concentrated on the forward roadway area before renovation to greater attention toward building facades, vegetation landscapes, and pedestrian interfaces on both sides of the street. This indicates that, under static streetscape image-viewing conditions, the visual attention pattern of the post-renovation images changed to some extent.

Specifically, the expansion of pedestrian space and the addition of resting facilities encouraged participants to allocate more visual attention to sidewalks and streetside landscapes on both sides of the street. Meanwhile, the increase in sidewalk area and the reduction of motorized vehicle lanes reduced the visual occupancy and interference of traffic-related elements such as vehicles. It should be noted that, because the post-renovation stimuli were design renderings, their image cleanliness, color representation, and dynamic disturbance elements may differ from those of the pre-renovation field photographs. Therefore, these changes should be interpreted as shifts in fixation patterns under the conditions of design-scheme representation, rather than directly interpreted as an enhancement of the attractiveness of the actual street environment.

Overall, the visual hotspots in the post-renovation images partially shifted from circulation-related elements, such as roadways and vehicles, toward historical buildings, vegetation, sidewalks, and streetside interfaces, suggesting a certain degree of reorganization in visual attention patterns. However, these findings primarily reflect changes in visual attention allocation under controlled static image-viewing conditions and should not be directly equated with improvements in real-world walking experiences.

### 3.2 Changes in fixation patterns before and after streetscape renovation

The results indicate (Appendix B) that the distribution of fixation across AOI elements changed systematically after renovation. In A1-1 and A1-2, historical signage, historical buildings, and vegetation became the core AOIs showing the clearest increases in attention, with marked rises in both total fixation duration and fixation count. By contrast, attention directed to residential buildings, obstructions, and vehicles generally declined, whereas sky, sidewalks/paving, and facilities increased only slightly or remained broadly stable. In A2-1, the major changes were concentrated in vegetation, sidewalks/paving, and facilities, all of which showed substantial increases in total fixation duration and fixation count, while attention directed to commercial buildings, obstructions, and the sky declined. In A2-2, vegetation, historical signage, and historical buildings were all strengthened simultaneously, together with increases in attention to sidewalks/paving and facilities, whereas attention directed to commercial signage, vehicles, the sky, obstructions, and residential buildings declined overall. In B1-1, the increase in historical signage was the most pronounced of all elements; vegetation and historical buildings also increased substantially, path-related elements such as sidewalks, roadways, and paving rose slightly, and attention directed to commercial signage, obstructions, and residential buildings declined significantly, with a slight decline also observed for the sky. Both B2-1 and B2-2 showed increased total fixation duration and fixation count for historical buildings and historical signage, together with greater attention to sidewalks and selected facilities, whereas vehicles, residential buildings, and obstructions declined. In B2-1, vegetation exhibited a mixed pattern, with fixation count increasing but total fixation duration declining slightly; in B2-2, vegetation increased in parallel with historical buildings and historical signage, while commerce-related elements generally declined modestly. In A3-1, vegetation and historical buildings showed the clearest post-renovation increases, with substantial rises in both total fixation duration and fixation count; facilities, roadways, and sidewalks also increased to some extent, whereas vehicles, commercial signage, and obstructions declined markedly, the sky declined slightly, and residential buildings changed little. This result indicates that the addition of vegetation and public facilities does not necessarily enhance the perception of historical and cultural characteristics. When newly added vegetation or facilities are located in the foreground of historical buildings or within major visual corridors, they may improve environmental comfort and encourage staying behavior, but they may also obstruct historical building facades and weaken the visual salience of the historical-building AOI. Consequently, participants’ attention may shift from historical and cultural elements toward environmentally supportive elements such as vegetation, paving, and facilities.

Therefore, the B3 segment should not be regarded as a general exception but rather as an important counterexample in this study. This finding suggests that the renovation of HCSs should not simply pursue increased greenery and additional facilities, but should prioritize the visibility of historical building facades, entrance interfaces, and cultural signage. Future design practices should achieve coordination between ecological comfort and cultural legibility by controlling plant height, reserving visual corridors, reducing foreground obstruction of facades, and adopting low-height, point-based planting strategies.

### 3.3 Changes in fixation on AOI elements before and after renovation

**Figure 4:**
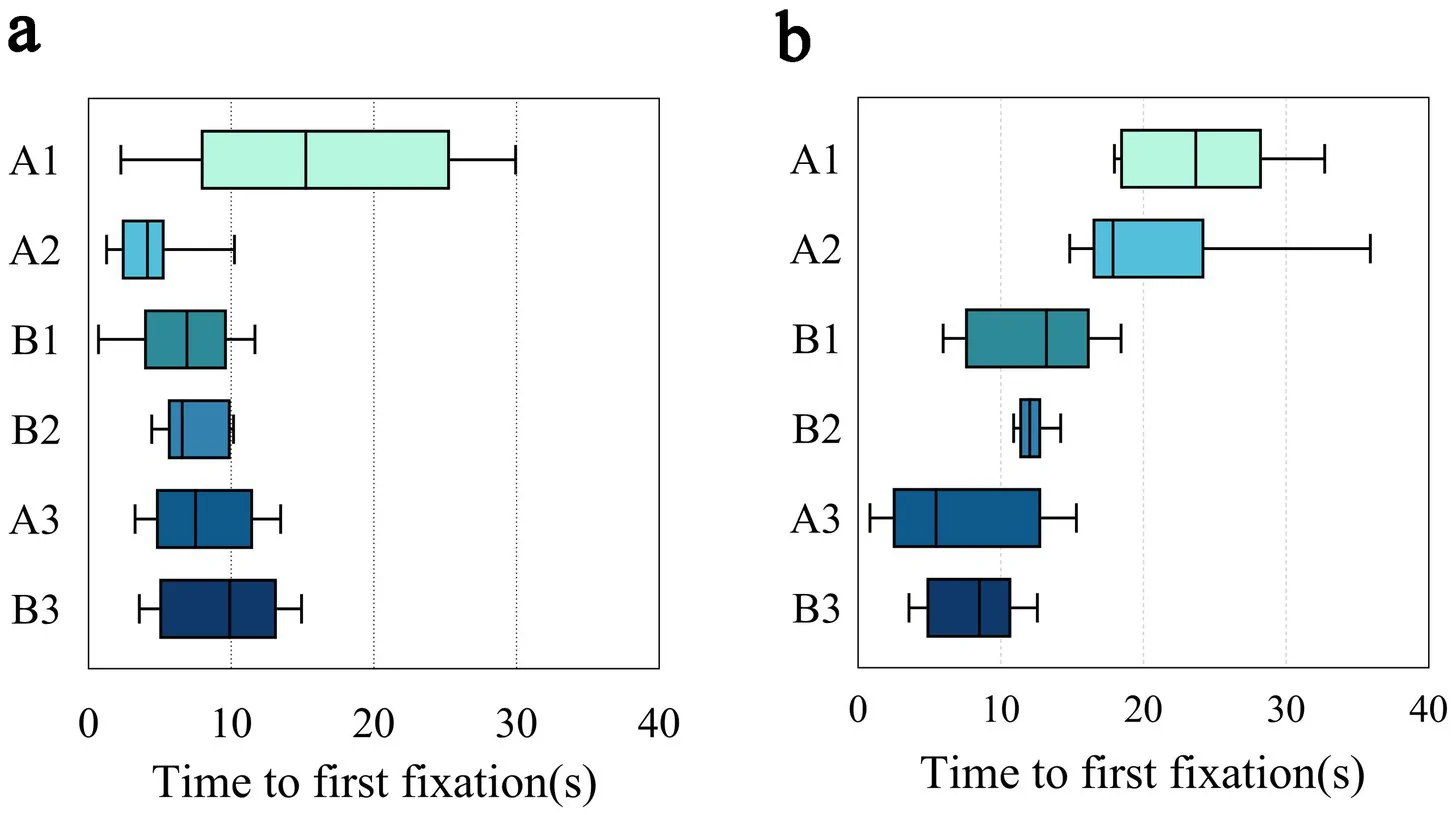
Proportion of Time to First Fixation in Pre-renovation Streetscapes. **(a)** Positive Streetscape Element Enhancement Group; **(b)** Negative Streetscape Element Optimization Group.

In this study, sky, vegetation, historical buildings, sidewalks, historical signage, and paving were classified as the Positive Streetscape Element Enhancement Group which was primarily used to analyze changes in visual attention toward elements associated with cultural expression, ecological landscape, and pedestrian environments after renovation. Roadways, residential buildings, commercial buildings, commercial signage, vehicles, and obstructions were classified as the Negative Streetscape Element Optimization Group which was mainly used to examine changes in the visual occupancy of traffic-, commercial-, and obstruction-related disturbance elements after renovation. By organizing the original streetscape data, the spatial proportion map of street elements before renovation was generated (Figure 4). Subsequently, changes in eye-tracking metrics of the two groups before and after renovation were compared to investigate the influence of streetscape renovation on visual attention allocation.

**Figure 5:**
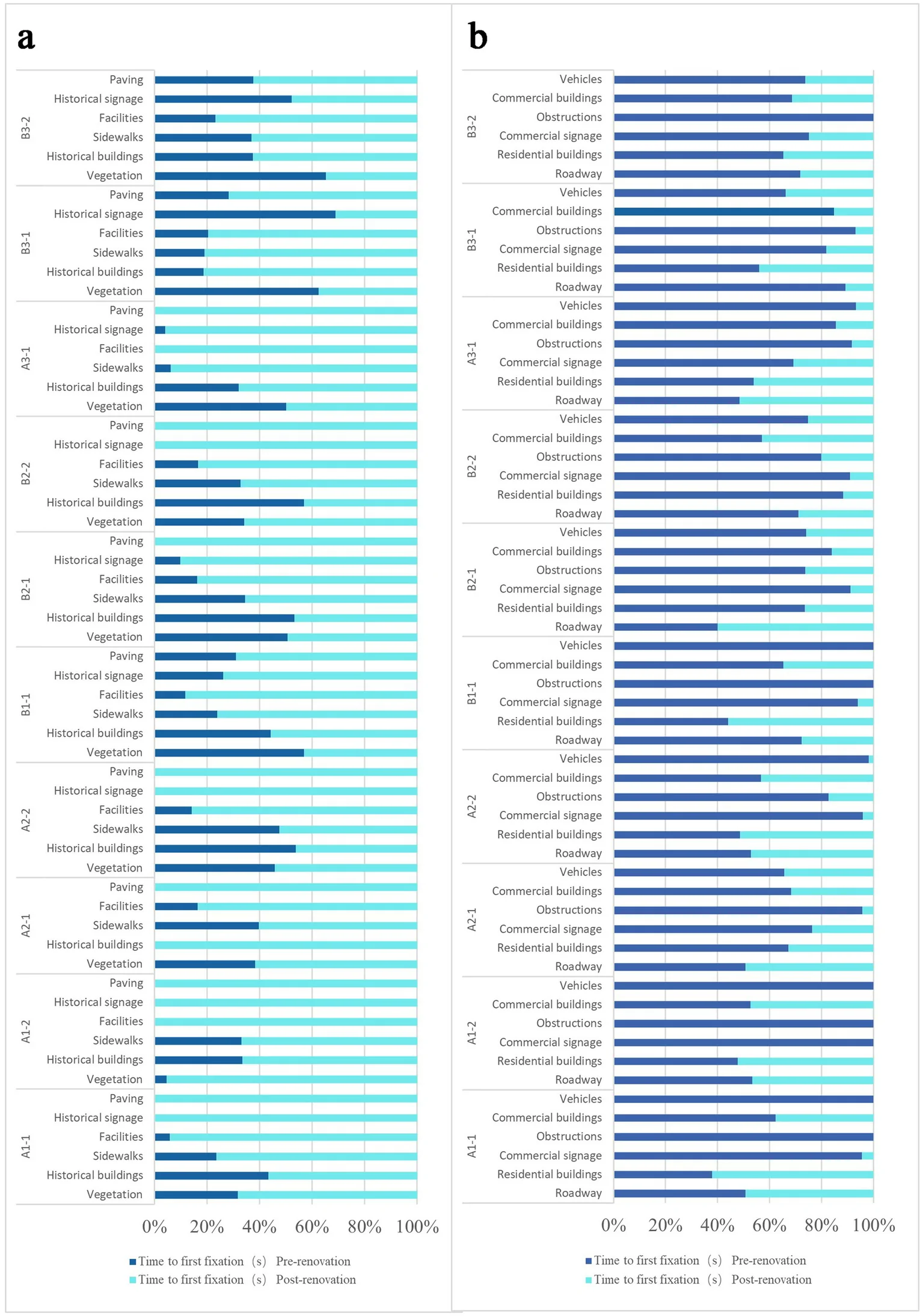
Comparison of streetscapes before and after renovation across street segments. **(a)** Positive Streetscape Element Enhancement Group; **(b)** Negative Streetscape Element Optimization Group.

After renovation, visual attention exhibited a relatively stable pattern characterized by decreases in negative elements and increases in positive elements. For example, total fixation duration and fixation count for historical signage and vegetation increased several-fold relative to the pre-renovation condition, indicating that attention directed to these optimized elements was substantially strengthened. Participants also spent longer on their initial fixation of these elements, suggesting that historical and cultural features and landscape elements had become the principal carriers of visual information in the street scene. In contrast, negative streetscape elements that were intentionally reduced in the renovation design generally showed decreases in fixation duration and fixation count after renovation. Correspondingly, traffic- and advertisement-related elements occupied a smaller proportion of visual attention in the post-renovation images. In particular, for roadway- and vehicle-related AOIs—which before renovation often attracted attention immediately upon scene entry—time to first fixation was significantly delayed after renovation, while total fixation duration declined, indicating that gaze was no longer overly concentrated on traffic-related disturbance and could instead be allocated more fully to historical and cultural elements. Taken together, these changes indicate that the renovated historical district achieved a shift in visual attention from negative to positive elements (Figure 5): Participants’ gaze was more evenly distributed among historical signage, buildings, vegetation, and other streetscape elements after renovation. At the image level, the street space exhibited a clearer visual order and hierarchical structure. However, whether these changes can further enhance actual users’ subjective evaluations of cultural atmosphere and spatial quality requires additional validation through field surveys or post-renovation subjective evaluation experiments.

A paired-sample t-test was conducted to compare the eye-tracking metrics between the Positive Streetscape Element Enhancement Group and the Negative Streetscape Element Optimization Group. The results showed that the relevant indicators differed significantly before and after renovation (*p* < 0.05) (Tables 1, 2), indicating that the allocation of visual attention in the post-renovation images underwent statistically significant changes. However, these findings primarily reflect changes in visual attention under static image-viewing conditions and should not be directly equated with increased attractiveness of the actual street environment.

**Table 1:** Comparison of eye-tracking metrics for positive streetscape elements before and after renovation.

Indicator	Paired samples (Mean ± SD)		Difference (Pre-Post)	*t*	*p*
	Pre-renovation	Post-renovation			
Time to first fixation	1.35 ± 1.54	3.19 ± 2.08	−1.84	−6.393	0.000***
First fixation duration	0.08 ± 0.09	0.12 ± 0.06	−0.05	−3.981	0.000***
Total fixation duration	0.61 ± 1.34	1.02 ± 1.00	−0.42	−2.902	0.005**
Fixation count	2.89 ± 6.45	5.82 ± 5.89	−2.93	−4.079	0.000***

**p < 0.01; ***p < 0.001.

**Table 2:** Comparison of eye-tracking metrics for negative streetscape elements before and after renovation.

Indicator	Paired samples (Mean ± SD)		Difference (Pre-Post)	*t*	*p*
	Pre-renovation	Post-renovation			
Time to first fixation	3.93 ± 2.15	1.70 ± 2.12	2.23	8.54	0.000***
First fixation duration	0.17 ± 0.09	0.05 ± 0.06	0.12	9.607	0.000***
Total fixation duration	0.57 ± 0.54	0.16 ± 0.24	0.41	6.056	0.000***
Fixation count	3.01 ± 2.71	0.98 ± 1.41	2.03	6.284	0.000***

***p < 0.001.

**Figure 6:**
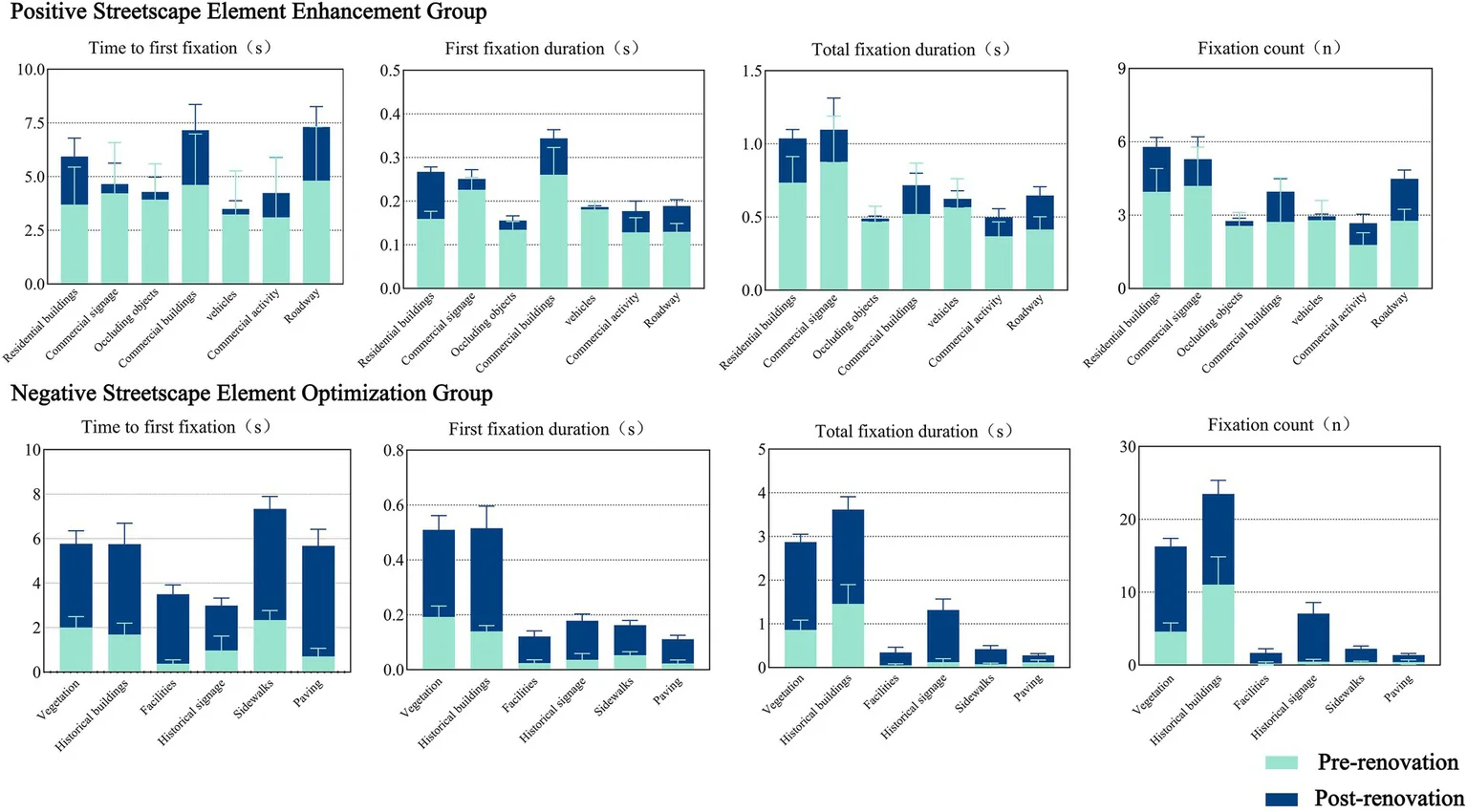
Overall comparison of streetscapes before and after renovation.

As shown in Figure 6, the Negative Streetscape Element Optimization Group placed greater emphasis on noise reduction and spatial order enhancement, thereby mitigating the excessive visual interference generated by vehicular traffic and commercial frontages. By contrast, the Positive Streetscape Element Enhancement Group strengthened the presence of greenery and historic elements, thereby improving the cultural salience and visual attractiveness of the HCSs and facilitating a transformation from a merely functional street environment into a historic streetscape characterized by contextual richness and mnemonic quality.

### 3.4 Changes in visual-structural indicators before and after streetscape renovation

**Figure 7:**
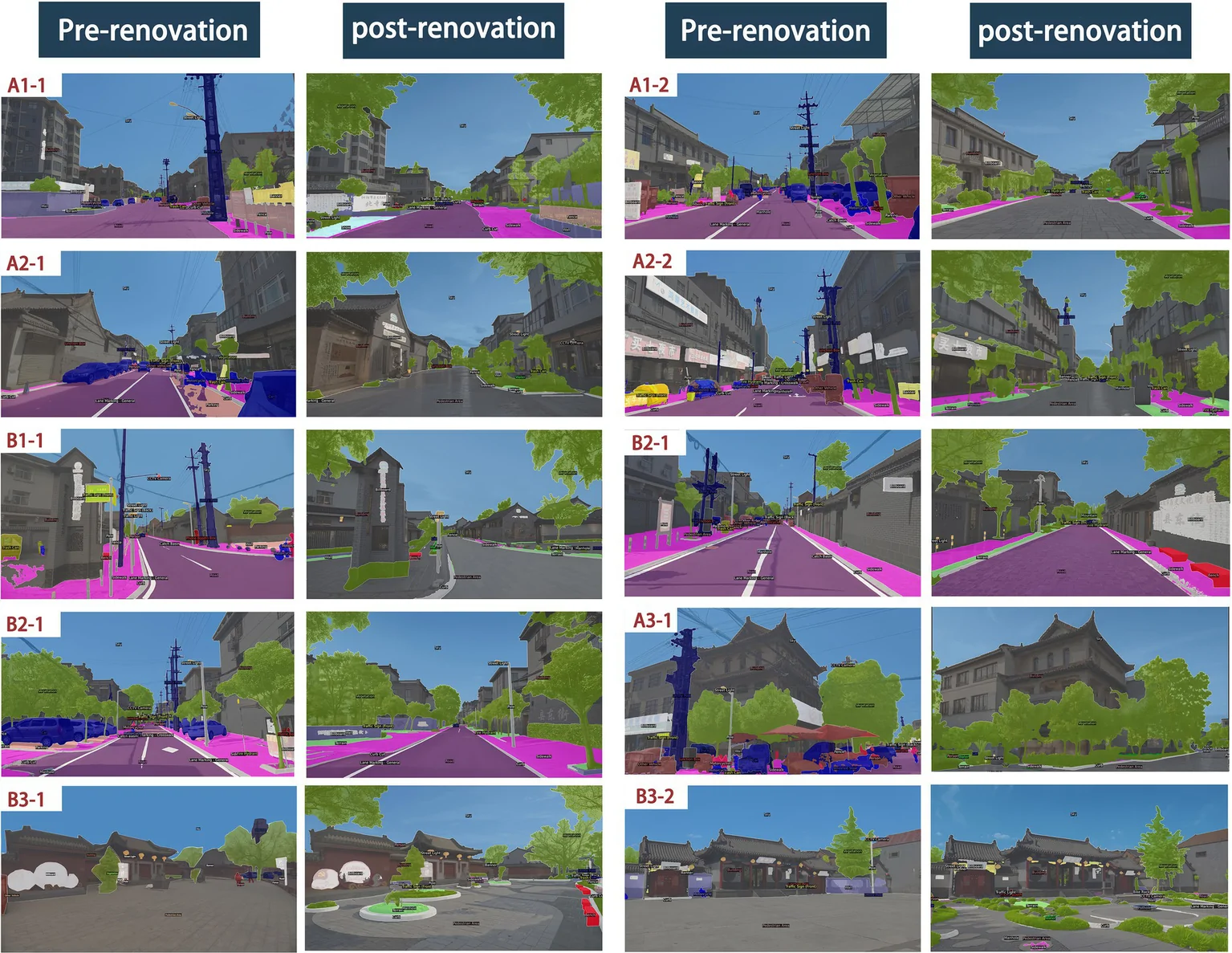
Semantic segmentation maps of typical streetscape nodes before and after renovation.

GVR, SO, PSP, SE, and MTIR were calculated according to Equations 1–5. After renovation, the streetscapes as a whole exhibited markedly improved visual-structural characteristics (Figure 7). The most prominent changes were observed in the GVR: A1-1 increased by approximately 25%, A1-2 by approximately 15%, and both A2-1 and A2-2 by more than 20%. Among these segments, A2-2, a commercial street segment, showed the largest increase, largely because additional tall trees were introduced without compromising the legibility of commercial signage, thereby improving shade provision and pedestrian comfort. In B1-1, a major scenic node distinguished by historical identity, SO increased by approximately 20%, which enhanced visual openness; pedestrian space was simultaneously expanded and motorized interference was reduced. B2-1 and B2-2 are located in the transitional zone between residential areas and historical streets; in these segments, moderate enclosure was emphasized while GVR and visual attention to historical buildings were adjusted or enhanced. The renovation of A3-1 focused on increasing the proportion of historical buildings within the visual field while also markedly expanding pedestrian space and green coverage. The B3 segment (B3-1 and B3-2) comprises plaza space in front of historical buildings. There, the original enclosure pattern was largely retained, while resting facilities and vegetation were added to improve spatial comfort and strengthen overall evaluation of the historical-street environment.

**Figure 8:**
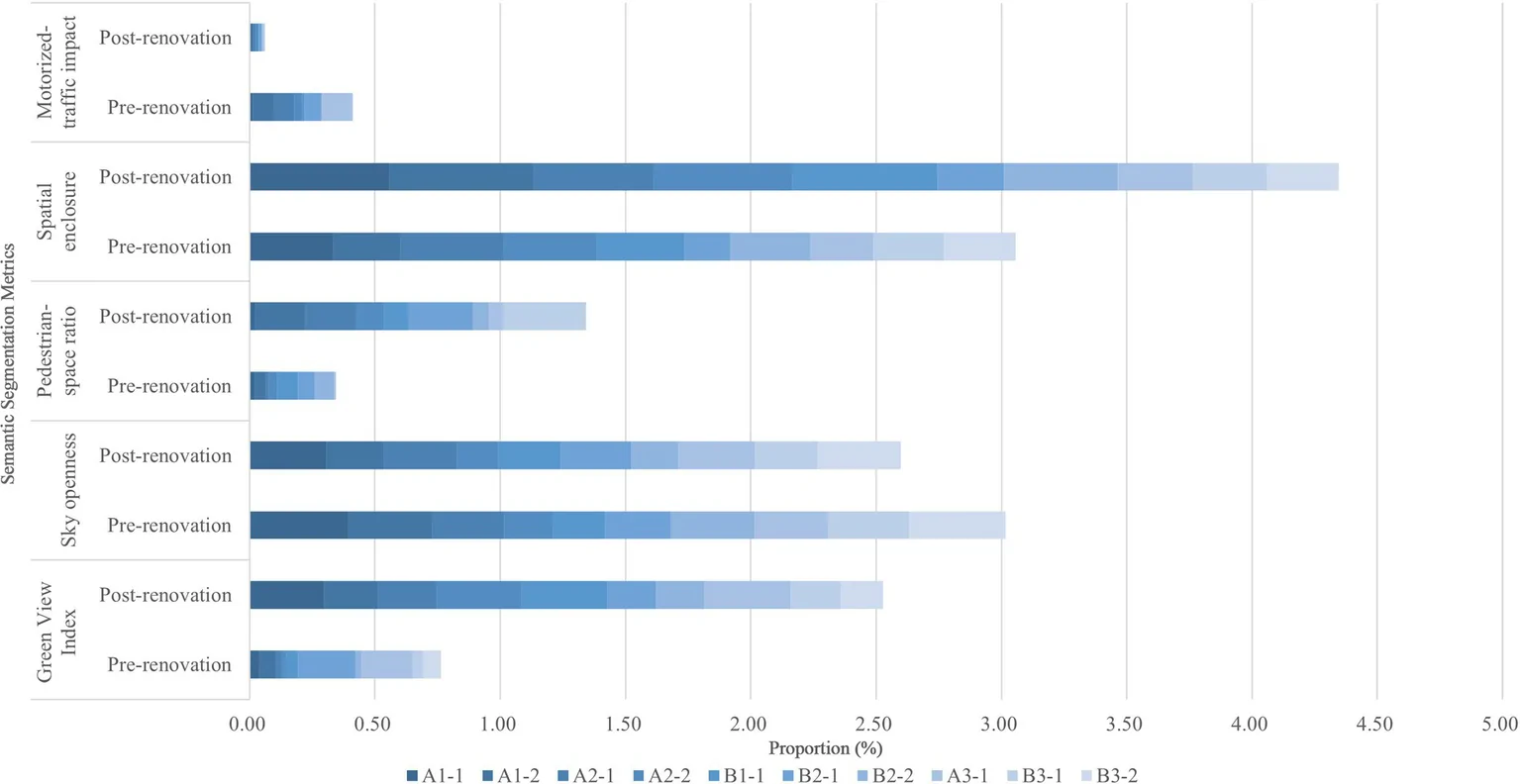
Comparison of semantic-segmentation data after renovation.

Comparison of the mean values of the visual-structural indicators further demonstrates the overall post-renovation changes (Table 3). The GVR increased from 7.6% before renovation to 25.3% after renovation, representing an increase of approximately 17.7 percentage points and about 3.3 times the pre-renovation value. It should be noted that, because the post-renovation images were design renderings, the substantial increase in GVR reflects not only the increased vegetation configuration proposed in the renovation scheme but may also have been influenced by the rendering style, image cleanliness, and vegetation representation methods. Therefore, this indicator should be regarded as a reference for visual-structural changes at the design stage rather than being directly equated with the actual GVR change after construction in the built environment. The PSP rose from 3.4 to 13.4%, indicating a substantial expansion of usable pedestrian space. The MTIR declined from 4.1 to 1.2%, showing that motor vehicles occupied the street space to a much lesser extent after renovation. SO decreased slightly from 30.2 to 26.0%, but remained within a broadly reasonable range. SE increased from 30.6 to 43.5%, suggesting that the post-renovation images exhibited a stronger sense of spatial coherence and formality at the visual level. Across street segments, the renovation followed a common logic of adding greenery, optimizing pedestrian space, controlling motorized traffic, and guiding visual attention, although differentiated strategies were adopted according to local function and spatial condition. The A1 and A2 segments focused primarily on supplemental planting and the widening of pedestrian space, together with the compression of motor-vehicle interfaces. In commercial segments such as A2-2, tall trees were used to reconcile shade provision with signage visibility. As a signage-display area, the B1 segment enhanced sign legibility by increasing visual openness and pedestrian capacity. The B2 segment increased greenery under conditions of moderate enclosure while reinforcing attention to historical buildings. The A3 segment increased the visual proportion of historical buildings and improved the slow-traffic system. In the B3 segment, additional facilities and vegetation were introduced without substantial changes to the existing interface pattern. However, the decline in attention to the historical-building AOI suggests that the arrangement of vegetation and public facilities should be further coordinated with the visibility of historical buildings in such spatial contexts (Figure 8).

**Table 3:** Comparison of visual-structural indicators derived from semantic segmentation before and after renovation.

Location	GVR		SO		PSP		SE		MTIR	
	Before	After	Before	After	Before	After	Before	After	Before	After
A1-1	0.039	0.294	0.393	0.305	0.020	0.022	0.332	0.557	0.012	0.000
A1-2	0.067	0.218	0.334	0.229	0.044	0.198	0.269	0.376	0.085	0.000
A2-1	0.019	0.233	0.289	0.293	0.011	0.206	0.411	0.578	0.080	0.000
A2-2	0.022	0.339	0.194	0.163	0.035	0.108	0.372	0.395	0.031	0.000
B1-1	0.046	0.343	0.206	0.250	0.083	0.100	0.350	0.579	0.010	0.000
B2-1	0.229	0.193	0.264	0.281	0.066	0.259	0.181	0.266	0.066	0.000
B2-2	0.024	0.194	0.335	0.189	0.078	0.062	0.321	0.454	0.004	0.000
A3-1	0.206	0.344	0.293	0.306	0.008	0.057	0.252	0.300	0.120	0.120
B3-1	0.042	0.200	0.325	0.249	0.000	0.329	0.281	0.394	0.001	0.000
B3-2	0.071	0.168	0.383	0.333	0.000	0.000	0.285	0.387	0.003	0.003
Mean	0.076	0.253	0.302	0.260	0.034	0.134	0.306	0.475	0.041	0.012

## 4 Discussion

### 4.1 Post-renovation heat maps indicate a reorganization of fixation patterns

After renovation, participants’ gaze shifted from the center of the roadway toward both sides of the street, becoming more concentrated on key elements such as building facades, historical signage, and vegetation. It should be noted that the present study was based on static streetscape images; therefore, the findings cannot be regarded as fully equivalent to actual pedestrian experience. This pattern of attentional redistribution is consistent with findings from previous evaluations of street renewal, which have shown that, as pedestrian space is strengthened and interface order is improved, visual attention tends to shift from the forward movement corridor toward façade details, signage nodes, and elements that support lingering and engagement (Fang et al., 2024). The post-renovation heat-map patterns observed here are therefore consistent with recent evidence that combines heat maps and preference assessment to evaluate the effectiveness of street renewal (Liu and Neisch, 2025).

### 4.2 Attention was redirected toward positive elements after renovation

Typical street segments such as A1-1 exhibited significant increases in both fixation duration and fixation count for historical signage, historical buildings, and vegetation, whereas attention directed to visually cluttered interfaces—such as residential buildings, obstructions, and disordered commercial activity—declined. This redistribution of attention, characterized by gains in positive elements and losses in negative elements, accords with common findings in eye-tracking studies of cultural heritage and historical character: once symbolic façade details become clearer and their recognition costs are reduced, observers are more likely to form stable fixations and less likely to expend attention on high-contrast but semantically uninformative distractors. The results of the B3 segment further demonstrate that increased greenery does not necessarily produce positive visual effects under all circumstances. After renovation, environmentally supportive elements such as vegetation, sidewalks, and paving in B3-1 and B3-2 received greater visual attention, whereas the total fixation duration and fixation count for the historical-building AOI decreased significantly. This indicates that when plant scale, planting location, or public facility arrangement affects the visibility of historical building facades, increased greenery may weaken the visual dominance of cultural elements. Therefore, greening design in the renewal of HCSs should follow the principle of “greening without obscuring historical features” by controlling plant height, reserving visual corridors toward historical buildings, and optimizing the arrangement of foreground facilities, thereby achieving a balance between ecological comfort and cultural legibility (Yang et al., 2024; Ma et al., 2021).

### 4.3 Integrating semantic segmentation and eye tracking provides strong evidence for evaluating streetscape renovation

A substantial body of research based on streetscape imagery and deep learning has shown that the GVR is significantly associated with walking behavior, physical activity, and perceived environmental quality, and that semantic segmentation is widely used to quantify street vitality and walkability (Li et al., 2023). Recent studies have also begun to combine streetscape-element recognition with eye-tracking data in order to explain how individual elements become attentional foci and thereby influence preference (Wang et al., 2022). By linking the visual-structural changes revealed by semantic segmentation with the attentional changes captured through eye tracking, it becomes possible to evaluate more comprehensively whether renovation has strengthened the visibility of cultural information. Semantic segmentation captures the outcomes of environmental transformation, whereas eye tracking reveals the allocation of attentional resources. When the directions of these two changes are consistent, it indicates that the visual-structural changes and visual attention allocation changes in the post-renovation images exhibit a relatively strong correspondence. Conversely, when inconsistencies occur between them, it suggests that design evaluation should not rely solely on quantitative indicators, but should further consider spatial-organization issues such as element location, visual obstruction, and interface order, which represent key challenges in historical street renovation.

These results indicate that, under static streetscape image-viewing conditions, the post-renovation images generally guided participants to allocate more visual attention toward elements related to cultural expression and pedestrian environments. However, this finding still requires further validation by controlling for differences in image types and examining visual perception under real street-use contexts (Kang et al., 2023). In the post-renovation images, positive streetscape elements received greater visual attention, whereas fixation on negative elements decreased. This pattern is broadly consistent with the attention resource allocation model in environmental psychology, which suggests that, under more organized visual-environment conditions, observers may preferentially allocate attention to information-rich positive elements, potentially facilitating deeper information processing and more efficient environmental perception.

### 4.4 Study limitations

This study has several limitations. First, the pre- and post-renovation stimuli consisted of field photographs and design renderings, respectively. These two types of images inherently differ in lighting, color saturation, image cleanliness, visual noise, and the representation of dynamic elements such as pedestrians and vehicles. Therefore, the eye-tracking results may have been jointly influenced by differences in image type and the renovation interventions. Second, the participants were predominantly young adults with higher education backgrounds. Although the sample included a small number of middle-aged and non-student participants, its representativeness of actual street users, such as older residents, local business operators, non-local visitors, and families, remains limited. Third, the renovation schemes were developed by the research team based on preliminary eye-tracking data and expert opinions, while AOI delineation, image selection, and interpretation of the results were also conducted by the research team. This overlap between the roles of designers and evaluators may have introduced interpretive bias. Fourth, the results for the B3 segment showed that the addition of vegetation and public facilities may weaken the visual salience of historical buildings, indicating that the renovation of HCSs should not simply pursue increased greenery but should also consider the visibility of historical buildings, visual corridors, and the control of foreground obstructions.

Future research could further improve the reliability and applicability of the findings by introducing a control group using “unrenovated renderings,” standardizing image brightness and color parameters, involving independent evaluators, recruiting more diverse participant groups, and conducting post-occupancy evaluations in actual built environments.

## 5 Conclusion

Streetscape renovation reconstructed attentional patterns at the level of landscape-related visual behavior. Heat-map comparisons showed that, before renovation, high-attention areas were concentrated in the central forward roadway band, whereas after renovation high-intensity regions expanded toward the interfaces on both sides of the street and more frequently covered building facades and other streetscape elements along the corridor. Attention thus shifted from a circulation-oriented pattern to an interface-information-oriented pattern, indicating that under static image-viewing conditions, the organization of visual attention and the pathways of information acquisition differed between pre- and post-renovation images.

At the streetscape level, most scenes in segments A1, A2, A3, B1, and B2 showed overall increases in total fixation duration and fixation count for culturally relevant elements, particularly historical signage and historical buildings. These increases were often accompanied by simultaneous gains in vegetation, sidewalks, paving, public facilities, and other elements associated with the pedestrian interface. By contrast, vehicles, obstructions, commercial signage, and the sky declined, or declined significantly, across multiple scenes. Paired-sample t-tests further showed that the key eye-movement indicators differed significantly before and after renovation (*p* < 0.05), thereby providing statistical support for the stability of the observed redistribution of attention. However, the decreased attention to the historical-building AOI in the B3 segment indicates that the shift in visual attention after renovation did not consistently correspond to enhanced attention toward historical and cultural elements across all street segments. Therefore, vegetation and facility arrangements should be carefully designed to avoid obstructing the visibility of historical buildings.

Changes in the visual-structural indicators were corroborated by the eye-tracking results. After renovation, the GVR and PSP increased significantly, whereas the MTIR declined. The overall visual structure of the streetscape therefore shifted in an optimized direction and was consistent with the redistribution of attention toward positive elements and away from negative elements, thereby enhancing the traceability and reusability of the interpretation of renovation effects.

Overall, this study demonstrates that, under static streetscape image-viewing conditions, the landscape renovation schemes for HCSs in Qinyang City exhibited an overall tendency for visual attention to shift toward elements related to cultural expression, vegetation landscapes, and pedestrian environments. These findings provide quantitative references for subsequent scheme optimization and refined design practices. It should be noted that, because the pre- and post-renovation stimuli were field photographs and design renderings, respectively, the results may have been influenced by differences in image types. Therefore, the findings cannot be directly equated with the effects of renovation in the actual built environment and require further validation through same-type image comparisons, field experiments, and post-occupancy evaluations.

## Data Availability

The original contributions presented in the study are included in the article/Supplementary material, further inquiries can be directed to the corresponding authors.
