# Supplementary material for: Visual perception of landscape renovation in Qinyang’s historical and cultural streets: evidence from eye tracking of static streetscape images
Source: Front Psychol. 2026 Sep 4;17:1848298. doi: 10.3389/fpsyg.2026.1848298 (PMC13585656; doi:10.3389/fpsyg.2026.1848298)
Supplement: Supplementary file 1 [file Table_1.docx]

# Appendix A

Table A1. Comparison of AOI Annotation Consistency Results

| AOI Category | No. of Samples | No. of Annotations by Annotator 1 | No. of Annotations by Annotator 2 | No. of Agreements | Agreement Rate | Remarks |
| --- | --- | --- | --- | --- | --- | --- |
| Sky | 20 | 20 | 20 | 20 | 100.00% | Consistent |
| Vegetation | 20 | 20 | 20 | 20 | 100.00% | Consistent |
| Historical buildings | 20 | 16 | 16 | 20 | 100.00% | Consistent |
| Sidewalks | 20 | 20 | 19 | 19 | 95.00% | Consistent |
| Facilities | 20 | 17 | 14 | 17 | 85.00% | Minor discrepancies |
| Historical signage | 20 | 20 | 16 | 16 | 80.00% | Requires careful re-examination |
| Paving | 20 | 10 | 10 | 20 | 100.00% | Consistent |
| Roadway | 20 | 10 | 10 | 20 | 100.00% | Consistent |
| Residential buildings | 20 | 16 | 16 | 20 | 100.00% | Consistent |
| Commercial signage | 20 | 17 | 14 | 17 | 85.00% | Minor discrepancies |
| Obstructions | 20 | 6 | 6 | 20 | 100.00% | Consistent |
| Commercial buildings | 20 | 15 | 15 | 18 | 90.00% | Minor discrepancies |
| Vehicles | 20 | 13 | 13 | 20 | 100.00% | Consistent |
| Commercial activity | 20 | 17 | 17 | 20 | 100.00% | Consistent |

Table A2. Summary of AOI Annotation Consistency Results

| Item | Value |
| --- | --- |
| Total sample size, N | 280 |
| Number of agreements | 267 |
| Observed agreement,Po | 0.9536 |
| Chance agreement,Pe | 0.6296 |
| Cohen's κ | 0.8746 |
| The overall agreement rate between the two annotators regarding AOI presence was 95.36%, with Cohen’s κ = 0.875, indicating a high level of consistency in category judgment. Discrepant items were discussed and reconciled to generate the final AOI annotations | |

# Appendix B

Tables. Eye-tracking data for street segments before and after renovation

## Site A1-1

| **AOI** | **Time to first fixation (Before/After, s)** | **Duration of first fixation (Before/After, s)** | **Total fixation duration (Before/After, s)** | **Fixation count (Before/After, n)** |
| --- | --- | --- | --- | --- |
| Sky | 0.56 / 0.75 | 0.52 / 0.10 | 0.13 / 0.28 | 1.25 / 2.38 |
| Vegetation | 1.21 / 2.61 | 0.10 / 0.22 | 0.44 / 1.58 | 2.34 / 9.84 |
| Historical buildings | 4.19 / 5.47 | 0.10 / 0.14 | 0.16 / 1.36 | 0.89 / 6.81 |
| Sidewalks | 2.40 / 7.76 | 0.03 / 0.06 | 0.09 / 0.16 | 0.51 / 1.16 |
| Facilities | 0.10 / 1.65 | 0.03 / 0.07 | 0.00 / 0.10 | 0.02 / 0.47 |
| Historical signage | 0.00 / 2.24 | 0.00 / 0.16 | 0.00 / 1.33 | 0.00 / 7.69 |
| Paving | 0.00 / 2.61 | 0.00 / 0.04 | 0.00 / 0.07 | 0.00 / 0.41 |
| Roadway | 7.01 / 6.83 | 0.16 / 0.11 | 0.39 / 0.29 | 4.70 / 2.13 |
| Residential buildings | 1.86 / 3.05 | 0.27 / 0.10 | 1.69 / 0.15 | 9.45 / 1.00 |
| Commercial signage | 3.68 / 0.17 | 0.19 / 0.01 | 0.18 / 0.01 | 0.92 / 0.03 |
| Obstructions | 2.93 / 0.00 | 0.17 / 0.00 | 1.05 / 0.00 | 5.81 / 0.00 |
| Commercial buildings | 5.39 / 3.26 | 0.15 / 0.05 | 0.27 / 0.12 | 1.66 / 0.94 |
| Vehicles | 1.26 / 0.00 | 0.13 / 0.00 | 0.01 / 0.00 | 1.26 / 0.00 |
| Commercial activity | 1.26 / 0.00 | 0.10 / 0.09 | 0.66 / 0.15 | 2.25 / 0.91 |

## Site A1-2

| **AOI** | **Time to first fixation (Before/After, s)** | **Duration of first fixation (Before/After, s)** | **Total fixation duration (Before/After, s)** | **Fixation count (Before/After, n)** |
| --- | --- | --- | --- | --- |
| Sky | 1.19 / 1.98 | 0.07 / 0.11 | 0.16 / 0.20 | 0.64 / 1.34 |
| Vegetation | 0.09 / 1.94 | 0.17 / 0.17 | 1.43 / 2.16 | 7.17 / 12.94 |
| Historical buildings | 0.35 / 0.69 | 0.09 / 0.13 | 1.02 / 2.94 | 5.34 / 18.94 |
| Sidewalks | 1.83 / 3.70 | 0.03 / 0.07 | 0.03 / 0.12 | 0.23 / 0.66 |
| Facilities | 0.00 / 2.71 | 0.00 / 0.04 | 0.00 / 0.05 | 0.00 / 0.34 |
| Historical signage | 0.00 / 1.78 | 0.00 / 0.13 | 0.00 / 1.29 | 0.00 / 7.91 |
| Paving | 0.00 / 3.32 | 0.00 / 0.06 | 0.00 / 0.13 | 0.00 / 0.84 |
| Roadway | 6.74 / 5.90 | 0.18 / 0.10 | 0.42 / 0.28 | 2.32 / 1.81 |
| Residential buildings | 4.67 / 5.13 | 0.18 / 0.09 | 0.93 / 0.25 | 4.96 / 1.75 |
| Commercial signage | 4.38 / 0.00 | 0.25 / 0.01 | 0.40 / 0.01 | 1.62 / 0.03 |
| Obstructions | 5.60 / 0.00 | 0.17 / 0.00 | 0.56 / 0.00 | 2.96 / 0.00 |
| Commercial buildings | 7.49 / 6.71 | 0.22 / 0.09 | 0.12 / 0.32 | 0.51 / 2.00 |
| Vehicles | 3.42 / 0.00 | 0.24 / 0.00 | 1.26 / 0.00 | 5.70 / 0.00 |
| Commercial activity | 3.51 / 2.80 | 0.15 / 0.05 | 0.15 / 0.08 | 0.98 / 0.50 |

## Site A2-1

| **AOI** | **Time to first fixation (Before/After, s)** | **Duration of first fixation (Before/After, s)** | **Total fixation duration (Before/After, s)** | **Fixation count (Before/After, n)** |
| --- | --- | --- | --- | --- |
| Sky | 2.08 / 1.30 | 0.16 / 0.08 | 0.57 / 0.13 | 2.87 / 0.81 |
| Vegetation | 0.62 / 1.00 | 0.23 / 0.15 | 0.45 / 2.22 | 1.96 / 13.50 |
| Historical buildings | 0.00 / 0.36 | 0.00 / 0.00 | 0.00 / 0.00 | 0.00 / 0.03 |
| Sidewalks | 3.79 / 5.77 | 0.08 / 0.12 | 0.02 / 0.28 | 0.13 / 1.78 |
| Facilities | 0.00 / 3.46 | 0.00 / 0.10 | 0.00 / 0.18 | 0.00 / 0.78 |
| Historical signage | 0.00 / 0.00 | 0.00 / 0.00 | 0.00 / 0.00 | 0.00 / 0.00 |
| Paving | 0.00 / 4.57 | 0.00 / 0.15 | 0.00 / 0.15 | 0.00 / 1.03 |
| Roadway | 7.09 / 6.90 | 0.15 / 0.13 | 0.21 / 0.35 | 1.25 / 2.25 |
| Residential buildings | 6.51 / 3.18 | 0.19 / 0.08 | 0.20 / 0.13 | 1.08 / 0.97 |
| Commercial signage | 8.49 / 2.62 | 0.29 / 0.15 | 1.69 / 1.30 | 8.85 / 7.41 |
| Obstructions | 5.38 / 0.24 | 0.18 / 0.00 | 0.62 / 0.00 | 3.09 / 0.06 |
| Commercial buildings | 5.88 / 2.72 | 0.38 / 0.16 | 2.25 / 0.53 | 11.66 / 3.44 |
| Vehicles | 0.23 / 0.12 | 0.00 / 0.00 | 0.00 / 0.00 | 0.00 / 0.00 |
| Commercial activity | 8.45 / 4.18 | 0.23 / 0.17 | 0.67 / 0.36 | 3.19 / 2.31 |

## Site A2-2

| **AOI** | **Time to first fixation (Before/After, s)** | **Duration of first fixation (Before/After, s)** | **Total fixation duration (Before/After, s)** | **Fixation count (Before/After, n)** |
| --- | --- | --- | --- | --- |
| Sky | 1.57 / 1.23 | 0.17 / 0.11 | 0.59 / 0.17 | 3.28 / 1.41 |
| Vegetation | 1.06 / 1.26 | 0.13 / 0.16 | 0.83 / 2.13 | 3.34 / 12.59 |
| Historical buildings | 2.37 / 2.04 | 0.18 / 0.17 | 1.76 / 2.35 | 8.92 / 11.72 |
| Sidewalks | 5.01 / 5.54 | 0.13 / 0.14 | 0.01 / 0.42 | 0.09 / 2.38 |
| Facilities | 0.00 / 3.77 | 0.00 / 0.13 | 0.00 / 0.21 | 0.00 / 0.97 |
| Historical signage | 0.00 / 3.45 | 0.00 / 0.29 | 0.00 / 1.00 | 0.00 / 3.66 |
| Paving | 0.00 / 5.87 | 0.00 / 0.12 | 0.00 / 0.27 | 0.00 / 1.66 |
| Roadway | 7.55 / 6.74 | 0.20 / 0.11 | 0.77 / 0.44 | 3.77 / 2.78 |
| Residential buildings | 6.39 / 6.74 | 0.18 / 0.15 | 0.80 / 0.51 | 4.42 / 3.25 |
| Commercial signage | 6.12 / 0.27 | 0.32 / 0.00 | 1.15 / 0.00 | 4.98 / 0.03 |
| Obstructions | 5.34 / 1.12 | 0.18 / 0.00 | 0.27 / 0.00 | 1.15 / 0.00 |
| Commercial buildings | 5.24 / 4.00 | 0.14 / 0.10 | 0.18 / 0.16 | 1.33 / 0.97 |
| Vehicles | 6.42 / 0.12 | 0.18 / 0.00 | 0.44 / 0.00 | 2.43 / 0.00 |
| Commercial activity | 4.89 / 0.07 | 0.20 / 0.01 | 0.34 / 0.01 | 1.68 / 0.03 |

## Site B1-1

| **AOI** | **Time to first fixation (Before/After, s)** | **Duration of first fixation (Before/After, s)** | **Total fixation duration (Before/After, s)** | **Fixation count (Before/After, n)** |
| --- | --- | --- | --- | --- |
| Sky | 1.99 / 1.23 | 0.06 / 0.10 | 0.21 / 0.12 | 1.00 / 0.84 |
| Vegetation | 3.51 / 2.65 | 0.09 / 0.17 | 0.22 / 1.28 | 1.00 / 6.81 |
| Historical buildings | 4.42 / 5.56 | 0.19 / 0.14 | 1.29 / 2.12 | 7.00 / 11.22 |
| Sidewalks | 1.81 / 5.77 | 0.06 / 0.16 | 0.12 / 0.35 | 1.00 / 1.81 |
| Facilities | 0.28 / 2.11 | 0.01 / 0.06 | 0.02 / 0.10 | 0.00 / 0.44 |
| Historical signage | 0.26 / 0.73 | 0.02 / 0.13 | 0.03 / 2.13 | 0.16 / 11.75 |
| Paving | 1.92 / 4.25 | 0.05 / 0.09 | 0.07 / 0.23 | 0.00 / 1.22 |
| Roadway | 3.22 / 1.23 | 0.10 / 0.13 | 0.28 / 0.50 | 1.00 / 2.75 |
| Residential buildings | 4.20 / 5.32 | 0.16 / 0.13 | 0.59 / 0.18 | 3.00 / 0.88 |
| Commercial signage | 1.86 / 0.12 | 0.19 / 0.00 | 2.23 / 0.00 | 11.00 / 0.00 |
| Obstructions | 3.60 / 0.00 | 0.14 / 0.00 | 0.71 / 0.00 | 4.00 / 0.00 |
| Commercial buildings | 0.86 / 0.46 | 0.51 / 0.01 | 0.08 / 0.01 | 0.19 / 0.09 |
| Vehicles | 0.20 / 0.00 | 0.00 / 0.00 | 0.00 / 0.00 | 0.00 / 0.00 |
| Commercial activity | 1.56 / 0.08 | 0.00 / 0.00 | 0.02 / 0.01 | 0.15 / 0.06 |

## Site B2-1

| **AOI** | **Time to first fixation (Before/After, s)** | **Duration of first fixation (Before/After, s)** | **Total fixation duration (Before/After, s)** | **Fixation count (Before/After, n)** |
| --- | --- | --- | --- | --- |
| Sky | 1.65 / 0.42 | 0.08 / 0.07 | 0.22 / 0.07 | 1.00 / 0.44 |
| Vegetation | 1.34 / 1.31 | 0.50 / 0.16 | 3.00 / 2.40 | 12.00 / 13.41 |
| Historical buildings | 1.51 / 1.32 | 0.09 / 0.16 | 0.10 / 2.03 | 0.24 / 10.88 |
| Sidewalks | 2.77 / 5.26 | 0.08 / 0.12 | 0.21 / 0.56 | 1.00 / 2.75 |
| Facilities | 0.15 / 0.78 | 0.01 / 0.02 | 0.01 / 0.02 | 0.00 / 0.09 |
| Historical signage | 0.33 / 3.05 | 0.03 / 0.17 | 0.03 / 1.61 | 0.00 / 8.91 |
| Paving | 0.00 / 2.56 | 0.00 / 0.03 | 0.00 / 0.08 | 0.00 / 0.44 |
| Roadway | 2.35 / 3.51 | 0.08 / 0.09 | 0.43 / 0.15 | 2.00 / 0.81 |
| Residential buildings | 3.12 / 1.12 | 0.17 / 0.17 | 0.92 / 0.42 | 5.00 / 2.41 |
| Commercial signage | 1.23 / 0.12 | 0.00 / 0.00 | 0.00 / 0.00 | 0.00 / 0.00 |
| Obstructions | 3.46 / 1.23 | 0.14 / 0.00 | 0.45 / 0.00 | 2.00 / 0.00 |
| Commercial buildings | 1.20 / 0.23 | 0.00 / 0.00 | 0.00 / 0.00 | 0.00 / 0.00 |
| Vehicles | 3.44 / 1.20 | 0.19 / 0.00 | 0.78 / 0.00 | 4.00 / 0.00 |
| Commercial activity | 1.86 / 0.60 | 0.19 / 0.02 | 0.19 / 0.05 | 0.55 / 0.25 |

## Site B2-2

| **AOI** | **Time to first fixation (Before/After, s)** | **Duration of first fixation (Before/After, s)** | **Total fixation duration (Before/After, s)** | **Fixation count (Before/After, n)** |
| --- | --- | --- | --- | --- |
| Sky | 2.81 / 1.15 | 0.12 / 0.06 | 0.31 / 0.09 | 2.00 / 0.53 |
| Vegetation | 1.61 / 3.10 | 0.16 / 0.17 | 0.68 / 1.25 | 3.00 / 7.41 |
| Historical buildings | 2.58 / 1.95 | 0.16 / 0.13 | 2.24 / 2.46 | 11.00 / 14.84 |
| Sidewalks | 2.83 / 5.82 | 0.08 / 0.13 | 0.18 / 0.52 | 1.00 / 3.09 |
| Facilities | 0.56 / 2.81 | 0.04 / 0.07 | 0.04 / 0.14 | 0.00 / 0.88 |
| Historical signage | 0.00 / 1.79 | 0.00 / 0.13 | 0.00 / 2.30 | 0.00 / 13.66 |
| Paving | 0.00 / 5.28 | 0.00 / 0.10 | 0.00 / 0.03 | 0.00 / 0.16 |
| Roadway | 3.03 / 1.23 | 0.13 / 0.15 | 0.75 / 0.57 | 4.00 / 3.50 |
| Residential buildings | 1.73 / 0.23 | 0.14 / 0.11 | 1.58 / 0.61 | 8.00 / 3.69 |
| Commercial signage | 2.30 / 0.23 | 0.10 / 0.00 | 0.16 / 0.00 | 1.00 / 0.00 |
| Obstructions | 4.78 / 1.20 | 0.13 / 0.00 | 0.26 / 0.00 | 2.00 / 0.00 |
| Commercial buildings | 2.83 / 2.14 | 0.16 / 0.08 | 0.22 / 0.00 | 1.00 / 0.00 |
| Vehicles | 3.56 / 1.20 | 0.14 / 0.00 | 0.32 / 0.00 | 1.00 / 0.00 |
| Commercial activity | 0.22 / 0.17 | 0.02 / 0.01 | 0.55 / 0.40 | 3.68 / 2.16 |

## Site A3-1

| **AOI** | **Time to first fixation (Before/After, s)** | **Duration of first fixation (Before/After, s)** | **Total fixation duration (Before/After, s)** | **Fixation count (Before/After, n)** |
| --- | --- | --- | --- | --- |
| Sky | 3.00 / 1.22 | 0.09 / 0.03 | 0.29 / 0.09 | 2.00 / 0.72 |
| Vegetation | 1.79 / 1.78 | 0.30 / 0.14 | 2.10 / 3.13 | 10.00 / 18.56 |
| Historical buildings | 1.03 / 0.18 | 0.21 / 0.19 | 2.56 / 3.33 | 12.00 / 20.09 |
| Sidewalks | 0.00 / 1.83 | 0.00 / 0.03 | 0.00 / 0.08 | 0.00 / 0.47 |
| Facilities | 0.00 / 4.09 | 0.00 / 0.07 | 0.00 / 0.17 | 0.00 / 1.13 |
| Historical signage | 0.00 / 0.24 | 0.00 / 0.01 | 0.00 / 0.02 | 0.00 / 0.19 |
| Paving | 0.00 / 10.47 | 0.00 / 0.03 | 0.00 / 0.03 | 0.00 / 0.16 |
| Roadway | 1.51 / 1.61 | 0.04 / 0.08 | 0.06 / 0.19 | 0.00 / 1.00 |
| Residential buildings | 3.52 / 3.02 | 0.13 / 0.10 | 0.34 / 0.32 | 2.00 / 2.38 |
| Commercial signage | 2.70 / 1.20 | 0.24 / 0.00 | 0.33 / 0.00 | 1.00 / 0.00 |
| Obstructions | 3.96 / 0.36 | 0.09 / 0.00 | 0.32 / 0.00 | 2.00 / 0.00 |
| Commercial buildings | 1.36 / 0.23 | 0.00 / 0.00 | 0.00 / 0.00 | 0.00 / 0.00 |
| Vehicles | 4.36 / 0.32 | 0.24 / 0.00 | 1.16 / 0.00 | 5.00 / 0.00 |
| Commercial activity | 0.00 / 0.00 | 0.00 / 0.00 | 0.00 / 0.00 | 0.00 / 0.00 |

## Site B3-1

| **AOI** | **Time to first fixation (Before/After, s)** | **Duration of first fixation (Before/After, s)** | **Total fixation duration (Before/After, s)** | **Fixation count (Before/After, n)** |
| --- | --- | --- | --- | --- |
| Sky | 2.40 / 1.19 | 0.07 / 0.07 | 0.23 / 0.10 | 1.00 / 0.47 |
| Vegetation | 4.10 / 2.47 | 0.10 / 0.16 | 0.29 / 1.70 | 2.00 / 9.72 |
| Historical buildings | 0.16 / 0.69 | 0.17 / 0.19 | 6.75 / 2.23 | 33.00 / 13.16 |
| Sidewalks | 1.39 / 5.93 | 0.03 / 0.21 | 0.07 / 0.87 | 0.00 / 3.94 |
| Facilities | 1.19 / 4.65 | 0.03 / 0.23 | 0.10 / 1.27 | 0.00 / 5.91 |
| Historical signage | 5.71 / 2.58 | 0.20 / 0.14 | 0.69 / 0.68 | 3.00 / 3.91 |
| Paving | 2.62 / 6.67 | 0.06 / 0.16 | 0.22 / 0.45 | 1.00 / 2.56 |
| Roadway | 1.32 / 0.16 | 0.00 / 0.00 | 0.00 / 0.00 | 0.00 / 0.00 |
| Residential buildings | 1.93 / 1.52 | 0.09 / 0.03 | 0.09 / 0.04 | 0.68 / 0.22 |
| Commercial signage | 1.62 / 0.36 | 0.00 / 0.00 | 0.00 / 0.00 | 0.00 / 0.00 |
| Obstructions | 1.62 / 0.12 | 0.00 / 0.00 | 0.00 / 0.00 | 0.00 / 0.00 |
| Commercial buildings | 3.62 / 0.65 | 0.00 / 0.00 | 0.00 / 0.00 | 0.00 / 0.00 |
| Vehicles | 2.35 / 1.20 | 0.00 / 0.00 | 0.00 / 0.00 | 0.00 / 0.00 |
| Commercial activity | 0.00 / 0.00 | 0.00 / 0.00 | 0.00 / 0.00 | 0.00 / 0.00 |

## Site B3-2

| **AOI** | **Time to first fixation (Before/After, s)** | **Duration of first fixation (Before/After, s)** | **Total fixation duration (Before/After, s)** | **Fixation count (Before/After, n)** |
| --- | --- | --- | --- | --- |
| Sky | 2.09 / 0.90 | 0.05 / 0.05 | 0.19 / 0.09 | 1.00 / 0.53 |
| Vegetation | 4.71 / 2.51 | 0.15 / 0.18 | 0.59 / 2.25 | 3.00 / 12.38 |
| Historical buildings | 0.26 / 0.43 | 0.21 / 0.20 | 6.67 / 2.77 | 32.00 / 16.78 |
| Sidewalks | 1.55 / 2.64 | 0.01 / 0.05 | 0.05 / 0.06 | 0.08 / 0.34 |
| Facilities | 1.54 / 5.09 | 0.12 / 0.18 | 0.35 / 0.67 | 2.17 / 3.47 |
| Historical signage | 2.46 / 2.25 | 0.08 / 0.11 | 0.35 / 0.36 | 1.00 / 1.78 |
| Paving | 2.51 / 4.17 | 0.12 / 0.10 | 0.60 / 0.19 | 3.00 / 1.03 |
| Roadway | 1.65 / 0.65 | 0.00 / 0.00 | 0.00 / 0.00 | 0.00 / 0.00 |
| Residential buildings | 3.06 / 1.62 | 0.08 / 0.14 | 0.21 / 0.26 | 1.00 / 1.25 |
| Commercial signage | 3.62 / 1.20 | 0.00 / 0.00 | 0.00 / 0.00 | 0.00 / 0.00 |
| Obstructions | 0.28 / 0.00 | 0.00 / 0.00 | 0.00 / 0.00 | 0.00 / 0.00 |
| Commercial buildings | 2.62 / 1.20 | 0.00 / 0.00 | 0.00 / 0.00 | 0.00 / 0.00 |
| Vehicles | 1.26 / 0.45 | 0.00 / 0.00 | 0.00 / 0.00 | 0.00 / 0.00 |
| Commercial activity | 0.00 / 0.00 | 0.00 / 0.00 | 0.00 / 0.00 | 0.00 / 0.00 |
